# Preliminary Study of Vision-Based Artificial Intelligence Application to Evaluate Occupational Risks in Viticulture

**DOI:** 10.3390/s25216749

**Published:** 2025-11-04

**Authors:** Sirio R. S. Cividino, Alessio Cappelli, Paolo Belluco, Fabiano Rinaldi, Lena Avramovic, Mauro Zaninelli

**Affiliations:** 1 Department of Human Science and Quality of Life Promotion, San Raffaele Telematic University, Via Val Cannuta 247, 00166 Rome, Italy; alessio.cappelli@uniroma5.it (A.C.); mauro.zaninelli@uniroma5.it (M.Z.); 2 Centro di Ricerca Studi dei Laghi, Via Vittor Pisani 8, 20100 Milano, Italy; 3 LWT3, Via Caduti di Marcinelle 7, 20134 Milano, Italy

**Keywords:** artificial intelligence, computer vision, occupational health and safety, risk assessment, viticulture, agricultural machinery, ergonomics, explainable AI

## Abstract

The agricultural sector remains one of the most hazardous working environments, with viticulture posing particularly high risks due to repetitive manual tasks, pesticide exposure, and machinery operation. This study explores the potential of vision-based Artificial Intelligence (AI) systems to enhance occupational health and safety by evaluating their coherence with human expert assessments. A dataset of 203 annotated images, collected from 50 vineyards in Northern Italy, was analyzed across three domains: manual work activities, workplace environments, and agricultural machinery. Each image was independently assessed by safety professionals and an AI pipeline integrating convolutional neural networks, regulatory contextualization, and risk matrix evaluation. Agreement between AI and experts was quantified using weighted Cohen’s Kappa, achieving values of 0.94–0.96, with overall classification error rates below 14%. Errors were primarily false negatives in machinery images, reflecting visual complexity and operational variability. Statistical analyses, including McNemar and Wilcoxon signed-rank tests, revealed no significant differences between AI and expert classifications. These findings suggest that AI can provide reliable, standardized risk detection while highlighting limitations such as reduced sensitivity in complex scenarios and the need for explainable models. Overall, integrating AI with complementary sensors and regulatory frameworks offers a credible path toward proactive, transparent, and preventive safety management in viticulture and potentially other high-risk agricultural sectors. Furthermore, vision-based AI systems inherently act as optical sensors capable of capturing and interpreting occupational risk conditions. Their integration with complementary sensor technologies—such as inertial, environmental, and proximity sensors—can enhance the precision and contextual awareness of automated safety assessments in viticulture.

## 1. Introduction

The viticulture sector, as a specialized branch of agriculture, continues to be one of the most hazardous work environments worldwide despite significant advancements in mechanization and automation [1,2,3,4,5,6,7].

Occupational injuries and fatalities in agriculture remain alarmingly high due to exposure to unpredictable environmental conditions, heavy machinery, chemicals, and physically demanding tasks. As the global demand for food production intensifies in response to population growth and climate change, the need for proactive safety management strategies becomes increasingly urgent. In the context of viticulture, this entails moving beyond reactive approaches—focused solely on accident reporting or compliance monitoring—toward anticipatory systems capable of identifying and mitigating risks before incidents occur. Such proactive safety management integrates continuous monitoring, data-driven risk prediction, and worker engagement to foster a preventive safety culture. By leveraging digital tools, including computer vision and AI-based analytics, vineyard operations can detect early signs of unsafe behaviors or environmental hazards, thereby reducing injury rates and ensuring regulatory compliance in dynamic agricultural settings [4,5,6,7,8,9].

Artificial Intelligence (AI) is emerging as a transformative technology across various domains, including agriculture, where it is reshaping traditional practices through precision farming, yield prediction, soil health monitoring, and pest control [10,11,12,13,14,15,16,17,18]. More recently, AI is also being explored for its potential to enhance occupational health and safety (OHS) through intelligent risk assessment systems. These systems leverage machine learning (ML), computer vision, and sensor networks to detect hazardous patterns, forecast high-risk scenarios, and support real-time decision-making for accident prevention [5,10,11,19,20,21,22]. AI applications have demonstrated relevance across several key operational domains. These include canopy management optimization through image-based leaf area estimation, yield prediction using multispectral and thermal imagery, and automated pest and disease detection leveraging convolutional neural networks trained on large-scale vineyard datasets. Moreover, AI-driven machinery navigation and precision spraying systems have significantly improved task accuracy and reduced pesticide overuse by up to 30–40%, according to recent precision agriculture studies [6,7,23,24]. Vision-based AI systems also enable real-time ergonomic assessment of workers’ posture during pruning, harvesting, and grape selection operations, supporting preventive occupational health management. Collectively, these advances illustrate how AI not only supports decision-making in vineyard management but also contributes to measurable improvements in productivity, safety, and environmental sustainability [15,16,17,18,25].

In the specific context of viticulture, AI has shown significant potential in several critical operations, such as canopy management, yield estimation, and disease detection through computer vision and multispectral imaging. Moreover, AI-driven systems have improved the precision of spraying, harvesting, and post-harvest grape sorting, contributing to a measurable reduction in pesticide use and ergonomic strain on workers [15,16,17,18,25].

However, applying AI in safety-critical agricultural environments introduces a new set of methodological and ethical challenges. Unlike conventional risk assessment approaches, grounded in deterministic models and statistical inference, AI systems are characterized by probabilistic behavior and often lack transparency in their decision-making processes. Their outputs can be influenced by data quality, model architecture, and environmental variability, which may complicate validation, regulatory compliance, and user trust.

Their behavior can be influenced by training data quality, model architecture, and environmental variability—all of which complicate the application of standards such as Safety Integrity Level (SIL) as defined by IEC 61508 [26]. Recent literature in functional safety emphasizes the need to reserve a portion of the dangerous-failure budget specifically for the probabilistic failure of AI components [13]. Moreover, models trained on non-representative data risk introducing biases or producing unexplainable outputs, thereby undermining trust and regulatory compliance [9,12]. To overcome these limitations, hybrid AI approaches combining methods such as latent class clustering, case-based reasoning (CBR), and artificial neural networks (ANNs) have been introduced to enhance both the reliability and interpretability of safety assessments, particularly in complex and data-scarce agricultural setting [14]. These systems can be based on complex models, nonlinear relationships between risk factors and incident severity, offering early-warning capabilities that surpass traditional rule-based systems. However, the deployment of such systems in the agricultural domain remains limited due to several barriers, including insufficient data infrastructure, limited digital literacy among workers, and a lack of standardized protocols for AI explainability and accountability. Responsible implementation frameworks—such as those recommended by the Alan Turing Institute—call for a socio-technical governance approach that embeds fairness, transparency, and safety across the AI lifecycle [9]. In the specific context of agriculture, and viticulture in particular, the occupational health risks are numerous and multifactorial, combining biomechanical, chemical, ergonomic, and psychosocial dimensions.

Epidemiological evidence consistently identifies agriculture as one of the highest-risk sectors in terms of work-related accidents and fatalities, with viticulture showing elevated incident rates due to repetitive tasks, prolonged awkward postures, pesticide handling, and machinery use [5,20]. Recent studies employing objective measures, such as accelerometry, have revealed alarmingly high rates of musculoskeletal pain among vineyard workers, particularly in the neck, upper back, and lower back regions. Standing work, repetitive manual activities above shoulder level, and inadequate rest–sleep cycles were significantly associated with pain severity, highlighting the critical role of posture and recovery in occupational health [19].

Chemical exposure further exacerbates safety concerns in viticulture. Chronic contact with phytosanitary products, particularly during preparation and application phases, has been linked to acute toxicological syndromes and long-term pathologies such as respiratory disorders, neurological syndromes, and even reproductive health issues [20]. Despite regulatory efforts, inappropriate use of personal protective equipment (PPE) persists, often due to misperceptions of risk or organizational limitations. Research indicates a mismatch between perceived and actual risk, suggesting the need for cognitive–behavioral interventions and improved risk communication strategies within safety protocols [8]. Beyond physical and chemical risks, the organizational and psychological factors significantly influence safety behavior. Studies on safety climate in winegrowing regions such as Bordeaux underscore the impact of workers’ risk perception on their actual compliance with safety procedures. Notably, participatory safety behaviors—such as mutual aid and proactive hazard reporting—were found to correlate more strongly with positive safety outcomes than simple procedural adherence, especially in decentralized and family-run farm structures [11]. In viticulture production, risks are closely linked to specific work phases. Manual pruning and canopy training expose workers to repetitive strain and awkward postures; pesticide preparation and spraying involve high chemical exposure, while tractor driving and mechanical harvesting present mechanical and rollover hazards. Activities performed in cellars, such as must transfer or barrel cleaning, add confined-space and inhalation risks. These examples highlight the multifactorial nature of viticultural hazards and the importance of AI-based monitoring systems for early detection and prevention [4,5,7].

Within viticultural production, specific occupational and phytosanitary risks arise from several repetitive and exposure-intensive activities. Among these, manual pruning and canopy training are associated with high biomechanical strain due to sustained non-neutral postures and repetitive upper-limb movements. Pesticide preparation and spraying operations expose workers to significant chemical and dermal risks, often exacerbated by inadequate PPE use and limited ventilation in confined vineyard areas. Mechanical harvesting and tractor operation present additional hazards, including vibration exposure, rollover risk, and entanglement injuries linked to rotating implements [7,10,11,12,13,20].

Furthermore, tasks performed in cellars, such as must transfer, fermentation monitoring, and barrel cleaning, pose chemical inhalation and confined-space risks. These examples emphasize the multifactorial nature of viticultural hazards and highlight the potential value of AI-based systems for early risk detection and exposure quantification through visual and sensor data integration [6,7,10,11].

These findings advocate for an integrated approach to occupational safety in agriculture, combining real-time physical risk detection through AI with contextual data on behavioral, cognitive, and organizational factors. By incorporating biomechanical monitoring, safety climate assessments, and predictive AI models into a unified risk assessment framework, a more holistic and preventive safety culture can be established in viticulture and broader agricultural contexts.

For these reasons, the first aim of this work is to evaluate the coherence, reliability, and regulatory alignment of AI-based occupational risk assessment systems within agricultural settings, with a specific focus on viticulture. By comparing hazard classifications produced by an AI system with those provided by human safety experts across a range of operational scenarios—including manual tasks, environmental settings, and machinery use—this study evaluates the validity and practical readiness of AI technologies for proactive safety management in agriculture. Through the combined use of image-based risk detection, legally contextualized interpretation frameworks, and structured risk matrix methodologies, the research aims to assess the feasibility of deploying explainable AI models as decision-support tools in occupational risk governance.

Particular attention is given to identifying discrepancies that may undermine decision-making reliability in safety-critical contexts [1,2,8,9,10,13,14].

## 2. Materials and Methods

### 2.1. Dataset

A structured and annotated image dataset was used to support the evaluation of a modular AI-based risk classification system in viticulture. The dataset comprised 203 real-world images collected from 50 winegrowing companies in Northern Italy. Stratified sampling ensured coverage of diverse operational settings and risk conditions across three domains:

- Manual work activities (*n* = 53)
- Workplace environments (*n* = 100)
- Agricultural machinery (*n* = 50)

The number of images per category reflects both the availability of scenarios in the field and the specific inclusion criteria required for each domain. Static environments were easier to capture and annotate, while scenes involving dynamic human activity or machinery operation demanded visual clarity, active task execution, and identifiable risk elements. Each image was labeled with relevant risk factors and assessed by both the AI system and human experts to allow systematic comparison. Prior to annotation, all images were anonymized to remove faces, logos, and geolocation metadata. Informed consent was obtained from all identifiable subjects.

### 2.2. Expert Panel

An expert panel was composed of three professionals specializing in occupational health and safety. The panel included two Occupational Safety Managers (RSPP) and one Environmental and Workplace Prevention Technician. These professionals routinely conduct safety inspections, perform risk evaluations, and oversee compliance with occupational health regulations within agricultural environments. The expert panel evaluated the same photographic dataset analyzed by the AI system, rather than the actual field environments. This approach ensured that both human and AI assessments were based on identical visual information, allowing a direct and unbiased comparison of classification outcomes. Their evaluations served as a qualitative reference for assessing the system’s practical applicability and the interpretability of its outputs within real-world operational contexts.

### 2.3. AI System Architecture and Processing Pipeline

**Figure 1:**
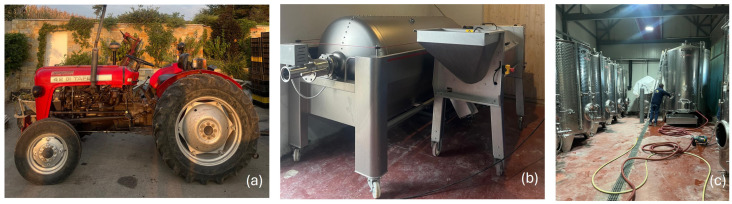
Examples of image data utilized for analysis in the study of occupational risk assessment in viticulture. (**a**) Agricultural machinery (tractor): Example of machinery commonly operated during vineyard activities, relevant for assessing mechanical and vehicular risk factors. (**b**) Workplace environment—grape reception area: Depiction of the grape processing zone, highlighting potential ergonomic and environmental hazards during grape unloading and preliminary processing). (**c**) Work activity—transfer from fermentation tanks: Manual operations involving liquid transfer from fermenters, used to evaluate physical effort, exposure to chemical agents, and confined space risks.

**Figure 2:**
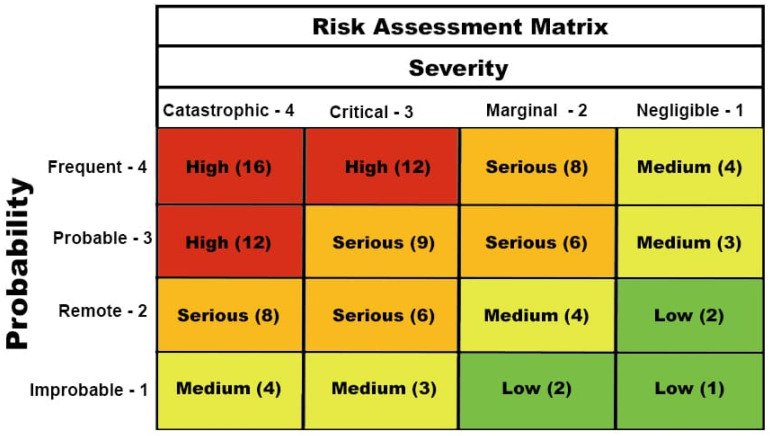
Example of risk assessment matrix.

The AI system followed a modular design and consisted of four sequential components:

1. Image Analysis: A convolutional neural network (CNN), trained on labeled agricultural datasets, was used to extract visual features such as operator posture, presence or absence of personal protective equipment (PPE), and machinery configuration. (Figure 1).
2. Risk Classification: Extracted features were classified into predefined occupational risk categories (e.g., mechanical, chemical, ergonomic, biological) using a supervised learning model. Class definitions followed standard safety taxonomies used in regulatory practice.
3. Regulatory Contextualization: Identified risk elements were interpreted against the Italian Legislative Decree 81/2008 [27] and relevant European directives (89/391/EEC [28], 89/654/EEC [29]) through a rule-based system. This module assessed whether visual conditions represented regulatory non-compliance.
4. Risk Matrix Evaluation: A traditional coarse risk matrix was applied, computing the product of probability (P) and severity (G) to assign each risk to one of four qualitative levels. Following Roos and Selitski (2023) [8], the matrix was constructed to ensure categorical clarity and minimize decision bias in safety-critical environments [21,22] (Figure 2).

### 2.4. Retrieval-Augmented Generation (RAG)

To support the generation of structured regulatory explanations within the Retrieval-Augmented Generation (RAG) module, we employed the ChatGPT system based on OpenAI’s GPT-4o model (June 2024 release). This choice was driven by the need for high linguistic accuracy, technical coherence, and reliable citation capabilities in a safety-critical context such as occupational risk assessment. Compared to alternative platforms such as Claude 3.7 Sonnet (Anthropic) or Gemini 2.0 Flash Thinking (Google DeepMind), GPT-4o demonstrated superior performance in legal and domain-specific language understanding, multilingual versatility, and fine-grained controllability over generation parameters. These features made it particularly suitable for producing verifiable, regulation-compliant content aligned with European occupational safety standards.

To enhance interpretability and regulatory traceability, a Retrieval-Augmented Generation (RAG) module was executed in parallel with the classification pipeline. This subsystem provided structured natural language explanations for each predicted risk, grounded in regulatory sources and procedural guidelines.

**(a)**  **Semantic Retrieval Layer**

This component employed a bi-encoder transformer model (Sentence-BERT), fine-tuned on occupational safety and legal corpora. The model performed dense vector retrieval from an indexed database consisting of

- National laws (e.g., D.Lgs. 81/2008);
- EU directives (e.g., 89/391/EEC, 89/654/EEC);
- Industry safety manuals and internal procedures.

For each hazard detected in an image, the system retrieved the top five most semantically relevant regulatory segments using cosine similarity in embedding space. Retrieved passages were filtered through a relevance threshold and subject to a token limit to ensure optimal input length for generation.

**(b)**  **Generative Reasoning Layer**

The retrieved texts were fed into a distilled BART encoder–decoder model, optimized for factual consistency. This model generated a structured justification for each risk prediction, comprising

- A concise description of the hazard;
- A normative explanation linking visual evidence to regulation;
- References to applicable standards or procedures.

Outputs were formatted as short, domain-specific explanatory paragraphs. The generated justifications were not used to influence classification outcomes but were appended to the final output to support expert auditing and regulatory validation.

**(c)**  **Pipeline Configuration and Data Privacy**

The regulatory corpus was preprocessed using sentence-level segmentation and anonymization. Indexing was performed using a FAISS-based vector store to support low-latency retrieval. The entire RAG workflow was executed locally, ensuring compliance with data protection regulations and maintaining full control over privacy-sensitive operations.

### 2.5. Evaluation of Assessment Concordance

To evaluate the consistency of AI-based risk classification, each image was independently assessed by the AI system and by human experts, and inter-rater agreement was measured. Agreement was deemed acceptable when both assigned the risk to the same category within the predefined risk matrix.

Cases of disagreement were examined to identify false negatives, overestimations, or underestimations by the AI model. All data were anonymized before analysis, and the study adhered to ethical standards and data protection regulations. The structured, tabular format of the dataset enabled systematic cross-comparison, providing insights into the operational robustness of the AI system and its potential for integration into conventional agricultural risk assessment workflow. In contrast to much of the existing literature, this study explicitly considers the potential sources of uncertainty and error associated with AI-based risk assessment systems. These include model overfitting, sensitivity to image quality, and variability in environmental conditions such as illumination and occlusion. By integrating these limitations into the methodological framework, the evaluation aims not only to measure AI performance but also to assess its reliability under real-world vineyard conditions. This approach contributes to a more transparent and realistic understanding of AI applicability in occupational safety, counteracting the common assumption of perfect algorithmic accuracy found in comparable works.

### 2.6. Statistical Analysis

The statistical analysis was designed to rigorously evaluate the level of agreement between risk classifications produced by the AI system and those provided by human safety experts. This comparison was carried out across three distinct domains relevant to viticultural operations: manual work activities, workplace environments, and agricultural machinery. To enhance clarity and methodological transparency, the following subsections describe in detail the specific statistical approaches adopted—namely, agreement assessment, error pattern analysis, and inferential comparison—each addressing a distinct aspect of the analytical framework.

### 2.7. Agreement Assessment

To quantify the consistency between AI and expert evaluations, the weighted Cohen’s Kappa coefficient was calculated for each domain. This metric was selected due to its suitability for ordinal data, reflecting the qualitative nature of the risk classes used in the study. High agreement values were interpreted as evidence of alignment between AI-based and expert-based assessments. Conversely, cases of disagreement were examined in detail to identify specific patterns of error, including

- False negatives: risks missed by the AI;
- Overestimations: risks rated higher than expert judgment;
- Underestimations: risks rated lower than expert judgment.

### 2.8. Error Pattern Analysis

To evaluate the consistency between AI outputs and expert judgment, an error pattern analysis was conducted. This analysis aimed to identify the conditions under which the AI system might fail to replicate expert assessments. Discrepancies between AI and human evaluations were systematically categorized according to the type of misclassification, with particular attention to errors in severity estimation. The categorization process considered contextual elements present in the images—such as machinery complexity, occlusions, worker posture, visibility of personal protective equipment (PPE), and environmental lighting—that could influence the model’s interpretation. This procedure provided a structured framework for identifying and describing recurrent error sources, which were subsequently examined in Section 3.

### 2.9. Inferential Comparison

To assess whether observed differences were statistically significant, inferential tests were applied:

- For Workplace zones, risk presence was dichotomized (present vs. absent), and the McNemar test was used to evaluate the symmetry of classifications between AI and human experts.
- For agricultural machinery, where risk was scored on a numerical scale, paired *t*-tests or Wilcoxon signed-rank tests (depending on data normality) were used to compare AI and expert ratings.
- For manual activities, a one-way ANOVA or Kruskal–Wallis test was conducted to explore whether agreement levels varied across risk categories (e.g., biomechanical, chemical, ergonomic).

This three-tiered statistical framework not only validated the robustness of the AI system in reproducing expert judgment but also exposed specific weaknesses. These findings reinforce the importance of targeted model improvements and underscore the need for transparent, explainable AI solutions in safety-critical domains like agriculture

## 3. Results

A series of statistical analyses was performed to assess the level of agreement between the AI system and human experts in classifying occupational risks across three domains: manual work activities, workplace environments, and agricultural machinery. The agreement was first evaluated using weighted Cohen’s Kappa, followed by an error distribution analysis. Where applicable, inferential statistics were used to further examine potential systematic discrepancies.

### 3.1. Inter-Rater Agreement

Inter-rater agreement was calculated using weighted Cohen’s Kappa (κ), with quadratic weights to reflect the ordinal structure of the 1–10 risk scale. Confidence intervals (95%) were estimated using 1000 bootstrap resamples. The results are summarized in Table 1.

The weighted Cohen’s Kappa values reported in Table 1 indicate an almost perfect agreement across all evaluated domains. The slightly lower value observed for agricultural machinery (κ = 0.939) reflects the higher variability and visual complexity typical of this category. Such performance demonstrates that the AI model can reliably reproduce expert judgment, particularly in static or semi-structured environments like workplace areas, supporting its potential integration into operational safety workflows.

### 3.2. Error Distribution

Classification errors by the AI system were categorized as underestimations, overestimations, or correct classifications, relative to expert ratings. The distribution of these errors across domains is presented in Table 2.

As shown in Table 2, underestimations and overestimations remain within an acceptable margin of error (<10%), confirming the internal consistency of the model. The predominance of false negatives in machinery-related images highlights how visual occlusions, lighting, and dynamic movements can influence detection accuracy. These findings underline the importance of integrating multimodal data sources—such as inertial or proximity sensors—to improve recognition performance in complex vineyard scenarios.

### 3.3. Inferential Analysis

To explore residual discrepancies between AI and expert assessments, domain-specific inferential tests were performed as detailed in Table 3.

- For workplace environments, risk scores were dichotomized (≥6 vs. <6) and compared using McNemar’s test, resulting in a symmetrical 2 × 2 contingency table (χ^2^ = 0.00, *p* = 1.000) [23].
- For agricultural machinery, the assumption of normality was rejected via the Shapiro–Wilk test (*p* < 0.001). Therefore, the Wilcoxon signed-rank test was applied, indicating no significant difference in paired ratings (W = 14, *p* = 1.000).
- For manual work activities, variation in AI–expert differences across risk types (biomechanical, chemical, ergonomic) were tested using the Kruskal–Wallis test, which revealed no significant heterogeneity (H = 2.35, *p* = 0.672).

The inferential tests summarized in Table 3 revealed no statistically significant differences between AI and expert assessments across all domains, confirming that the system performs comparably to human evaluators. This outcome reinforces the validity of the proposed methodology and suggests that, under standardized conditions, AI-based systems can provide consistent and replicable risk classifications suitable for integration in occupational safety management in viticulture.

Overall, these results demonstrate the methodological soundness of the proposed framework and its applicability to real-world vineyard contexts. The convergence between AI and expert assessments provides a strong basis for the subsequent discussion on operational reliability, explainability, and scalability of AI-based safety evaluation systems. These findings confirm that the proposed AI model achieves a performance level statistically equivalent to human evaluators, supporting its potential application as a decision-support tool in standardized occupational risk assessments. The overall stability of agreement metrics further indicates that the model generalizes effectively across heterogeneous visual contexts, suggesting robustness for deployment under real-world vineyard conditions.

## 4. Discussion

The evaluation of the AI system across manual work activities, workplace environments, and agricultural machinery showed a consistently high level of agreement with expert assessments, with weighted Cohen’s Kappa values between 0.939 and 0.963 and overall error rates below 14%. These findings align with prior studies that highlight the role of AI in domain-specific risk detection within precision agriculture (Izquierdo-Bueno et al., 2024) [1].

These results demonstrate that vision-based AI can serve not only as a classification tool but as an operational decision-support instrument within viticultural risk management. In comparison with prior research focused primarily on agronomic optimization (Bongiovanni & Lowenberg-DeBoer, 2004; Di Gennaro et al., 2025) [15,16], this study addresses a different domain—occupational safety—and provides one of the first empirical validations of an AI system benchmarked directly against certified safety experts in real-world vineyard conditions.

In the case of manual activities, particularly pruning, the system demonstrated strong performance in identifying ergonomic and biomechanical hazards under static conditions. This is consistent with the work of Li et al. (2020), who reported that RGB-based deep learning models can effectively estimate RULA scores with high real-time accuracy [3]. Nonetheless, the presence of occlusions—due to foliage, tools, or unfavorable camera positioning—led to missed detections in some cases. Such limitations indicate the potential benefit of integrating complementary data sources, such as inertial sensors or multispectral imaging, as suggested in recent smart viticulture literature (Izquierdo-Bueno et al., 2024) [1].

Recent studies confirm that multimodal fusion—combining visual and kinematic data—improves the detection of postural risk factors in field workers (Thamsuwan et al., 2024) [30]. Incorporating such approaches could enhance the robustness of AI-based ergonomic evaluations in viticulture, especially under dynamic task conditions.

In workplace environments, characterized by static and infrastructure-focused risk scenarios, the AI system maintained strong consistency with expert evaluations, aided by the integration of regulatory logic layers. The rule-based contextualization module allowed the system to interpret visual features in accordance with legal standards (e.g., D.Lgs. 81/2008, Directive 89/391/EEC, Directive 89/654/EEC), reducing ambiguity in safety classification. This aligns with Roos and Selitski (2023), who emphasized the importance of clear risk categorization thresholds and structured decision-making criteria in coarse risk matrix applications [8]. The introduction of a retrieval-augmented explanation mechanism (RAG) further strengthens this alignment by linking every AI-generated output to explicit regulatory sources. Such traceable reasoning is consistent with recommendations for trustworthy and explainable AI in safety-critical systems (Barredo Arrieta et al., 2020) [31]. In contrast, performance in the agricultural machinery domain was less robust, as evidenced by a higher rate of false negatives (8%). This was attributed to visual complexity, occlusions, and lighting variation, which affect the CNN’s sensitivity. These findings are consistent with the “Safety + AI” literature, which underscores the need to allocate a portion of the failure budget to algorithmic uncertainty in safety-critical contexts (Gheraibia et al., 2019) [2]. The system’s reliance on deep learning also introduces challenges related to model opacity, reinforcing the value of explainable AI frameworks. In this study, the parallel implementation of a Retrieval-Augmented Generation (RAG) module helped to mitigate these issues by producing regulatory justifications for each risk classification. This finding supports the argument that interpretability is a precondition for trustworthy AI deployment in occupational health domains Explainable outputs not only foster user confidence but also facilitate auditing processes required by the European AI Act and related safety governance frameworks (Leslie, 2019) [9]. The generated explanations, derived from legally grounded sources, increased interpretability without altering risk scores. Beyond performance metrics, the AI system consistently identified key risk exposures—such as prolonged standing and overhead tasks—that are widely recognized in the occupational health literature. Notably, De Lavor et al. (2024) found a 60% prevalence of lower back pain among vineyard workers, linked to both work-related and personal behavior patterns [4].

The consistency between AI predictions and such epidemiological evidence supports the potential integration of biomechanical data from wearable devices to provide a more complete risk profile and inform personalized preventive strategies. Several limitations emerged during the study, including the cross-sectional design, the restricted geographic scope (50 vineyard companies in Northern Italy), and the lack of longitudinal injury records. These factors limit the generalizability of the results and prevent assessment of the system’s long-term predictive value. Future research should address these constraints by incorporating time-series data, expanding geographical coverage, and validating the system in more heterogeneous agricultural environments. Its practical value will hinge on embedding the algorithms within a broader socio-technical framework that combines complementary sensor technologies, continuous model refinement, and transparent, explainable reasoning layers capable of justifying each risk attribution.

Equally essential is full alignment with emerging European regulatory requirements on trustworthy AI, so that stakeholders can audit performance, trace decisions, and assign accountability without eroding worker confidence (Leslie, 2019) [9,24].

### Integration with Sensor-Based Occupational Monitoring

Although this study focused primarily on image-based sensing, the integration of additional sensors can significantly enhance the reliability and interpretability of AI-driven occupational safety assessments.

- Inertial sensors (accelerometers, gyroscopes) can quantify worker posture, vibration exposure, and repetitive motion risk [30].
- Proximity and LiDAR sensors can detect unsafe distances between workers and machinery.
- Environmental sensors (temperature, humidity, gas concentration, particulate matter) can detect microclimatic or chemical risk factors [17,18].
- Wearable biosensors can monitor physiological stress indicators such as heart rate variability and fatigue, providing early warnings of overload conditions.

The fusion of visual data with these sensor inputs through multimodal AI frameworks has been demonstrated in recent precision agriculture and ergonomics studies to improve both detection accuracy and predictive validity [1,3,10,30,31].

Future developments of this system may involve integrated multi-sensor architectures capable of supporting continuous and context-aware risk monitoring through the synergistic fusion of heterogeneous data from optical, inertial, environmental, and physiological sensors, with the aim of enhancing the predictive accuracy, operational responsiveness, and real-time analytical capability of intelligent safety systems.

## 5. Conclusions

In conclusion, the integration of vision-based artificial intelligence into occupational risk assessment represents a promising avenue for enhancing the speed, consistency, and proactivity of safety management in viticulture. The results of this study, showing an almost perfect agreement between AI and expert evaluations, confirm that such systems can reliably replicate professional judgment under real-world vineyard conditions, providing a solid foundation for their operational adoption. To fully realize this potential, future implementations should adopt an iterative, human-centered design, wherein digital tools are developed to augment—rather than substitute—expert judgment and a participatory safety culture. By coupling technological innovation with robust governance frameworks and continuous user training, AI-enabled systems can support a paradigm shift from reactive risk mitigation to truly anticipatory prevention, offering a replicable model for other high-risk sectors. Nevertheless, it is essential to acknowledge the current limitations of AI technologies, including their reliance on high-quality training data, the opacity of deep learning models, and the risk of algorithmic bias—factors that may undermine user trust and hinder compliance with regulatory standards. Addressing these challenges will require future research efforts to focus on the development of explainable and hybrid AI systems that integrate multimodal data streams and emphasize collaborative human–AI decision-making. Such an approach is crucial to ensuring that applications in occupational risk management are not only technically effective but also transparent, resilient, and ethically aligned. Future research should also aim to apply the proposed AI-based risk assessment framework to specific vineyard operations that present particularly high-risk conditions, such as pesticide spraying, canopy management under slope conditions, and mechanical harvesting. These phases are characterized by a combination of biomechanical, chemical, and mechanical hazards that could benefit from targeted AI-based monitoring and predictive safety systems. From this perspective, the proposed framework not only advances methodological rigor in occupational safety research but also provides a scalable reference model for integrating trustworthy AI within the broader European strategy for sustainable and ethical digitalization of agriculture. Such focused applications would enable a more granular validation of model performance and further demonstrate the potential of AI to support proactive and domain-specific occupational risk prevention in viticulture. Future developments should explore hybrid sensing architectures that combine vision-based AI with wearable and environmental sensors, creating comprehensive, data-driven safety ecosystems for viticulture and other high-risk agricultural domains.

## Figures and Tables

**Table 1:** Agreement between the AI system and human experts across image domains.

Domain	Images (N)	Weighted Cohen’s κ	95% CI	Overall Agreement (%)
Work activities	53	0.957	[0.902–1.000]	94.3
Workplace areas	100	0.963	[0.933–0.983]	86.0
Agricultural machinery	50	0.939	[0.797–0.980]	86.0

**Table 2:** Distribution of AI classification errors.

Domain	Underestimations	Overestimations	Correct Classifications	Total
Work activities	0 (0.0%)	3 (5.7%)	50 (94.3%)	53
Workplace areas	5 (5.0%)	9 (9.0%)	86 (86.0%)	100
Agricultural machinery	4 (8.0%)	3 (6.0%)	43 (86.0%)	50

**Table 3:** Inferential tests comparing AI and expert assessments.

Domain	Statistical Test	Test Statistic	*p*-Value
Workplace areas	McNemar (risk ≥6 vs. <6)	χ^2^ = 0.00	1.000
Agricultural machinery	Wilcoxon signed-rank (scores 4–10)	W = 14	1.000
Work activities	Kruskal–Wallis (AI—Expert across risk types)	H = 2.35	0.672

## Data Availability

The data presented in this study are available on request from the corresponding author. The data are not publicly available due to privacy and ethical restrictions related to the anonymization of image datasets collected in operational vineyard environments.
